# Sub-Dissociative Ketamine Use in the Emergency Department for Treatment of Suspected Acute Nephrolithiasis: The SKANS Study

**DOI:** 10.51894/001c.7210

**Published:** 2019-03-04

**Authors:** Justin Grill, Caleb Bryant, Leonard Dunikoski, Zach Carrasco, Samuel J. Wisniewski, Kristen Price

**Affiliations:** 1 Department of Emergency Medicine Mercy Health Muskegon, MI; 2 Department of Pharmacy Mercy Health Muskegon, MI; 3 Statewide Campus System Michigan State University, East Lansing, MI

**Keywords:** opioid alternative, ketorolac/toradol, renal colic, sub-dissociative ketamine

## Abstract

**CONTEXT:**

Currently, there is no standard therapy for treatment of acute renal colic. With the increased scrutiny and controversy now surrounding opioids, the authors identified a need to investigate an alternative medication for pain control. As such, they sought to determine the efficacy of sub-dissociative (i.e., low) doses (0.3 mg/kg) of ketamine in providing Emergency Department (ED) patients acute pain management for renal colic secondary to nephrolithiasis.

**METHODS:**

After institutional review board (IRB) approval, the authors conducted a non-blinded, prospective clinical study. A convenience sample of n = 34 patients from the ED of a Western Michigan-based health system with suspected renal colic received one intravenous dose of ketorolac, 30 mg if over 50 kg body weight or 15 mg if under 50 kg In patients weighing greater than 50 kg, up to two doses of sub-dissociative ketamine were then given to further control pain. Pain was assessed at times 0, 30, 60, 90 and 120 minutes.

**RESULTS:**

There was a statistically significant pain reduction with administration of sub-dissociative ketamine, with 24 (69.2%) patients reporting an average reduction in pain score > 30% (t = 3.16, p = 0.004). Initial average pain scores for patients receiving sub-dissociative ketamine averaged 7.76 (SD = 2.55) on the 11-point verbal Pain Numeric Rating Scale. After a first dose of ketamine, patients’ average pain score was 3.56 (SD = 0.74) at 30 minutes. After two hours, patients’ average score was 2.56 (SD = 0.65), indicating that pain control was still effective over time with no statistically significant change in pain scores. Additionally, there was no statistically significant difference in pain reduction observed between genders (t = -0.192, p = 0.850).

**CONCLUSIONS:**

Based on these results, sub-dissociative ketamine may be considered a reasonable and effective supplemental non-opiate treatment option for suspected renal colic in otherwise healthy 18-70-year-old patients and could provide an effective alternative to traditional therapies. Further studies utilizing this methodology with larger, more generalizable samples are needed to further validate these findings.

## INTRODUCTION

Renal colic is an acute, severe, intermittent pain caused by a stone obstructing flow of urine in the genitourinary tract. Lodging of a stone within a ureter increases the hydrostatic pressure causing the urothelium to stretch and activate afferent autonomic pain fibers. These fibers originate from the Thoracic 10 through Lumbar 1 vertebrae levels resulting in known viscerosomatic reflex referred pain patterns to the flank, abdomen, groin, and/or genitalia.^1^ In the United States, acute onset renal colic represents 8.8% of all cases in the emergency departments (ED); with an estimated 1.2 million annual cases of nephrolithiasis.^1–3^ The lifetime risk of developing kidney stones for men in the US is 12% and 7% for women.^1,2^

Treatment opinions vary between providers, sources, and facilities. Steinberg et al. found that only 69% of renal colic patients received adequate analgesia while in the ED.^4^ Increasing incidence of renal colic has been associated with increased costs; in 2009, ED services associated with treatment of renal colic were over $5 billion.^5^ Further complicating matters, a specific standard for acute renal colic pain management in ED settings does not exist.^6^

Most evidence suggests non-steroidal anti-inflammatories are superior for analgesia with fewer side effects compared to opioids.^3,7–14^ Research has suggested concomitant opioid use with non-steroidal anti-inflammatory drugs (NSAIDs) was superior to either therapy alone; opioid usage was reduced by 49% when given with NSAIDs.^4,15,16^ Studies comparing acetaminophen with opioids and NSAIDs have yielded inconclusive results.^17,18^ Other alternatives such as acupuncture, antispasmodics, and fluid resuscitation have not been found to be superior than NSAIDs or opioids for analgesia.^12,14,17^ In light of the current opioid epidemic, there is also a compelling need for novel, non-narcotic pain medication for patients presenting to the ED with pain complaints. Sub-dissociative ketamine (SDK) may be a non-opioid alternative for acute pain management in emergent renal colic.^19,20^

During the past decade, SDK adjusted by patient weight (<1.0 mg/kg) has been recognized as an effective treatment option for acute pain for a variety of situations such as: wound dressing for burns, perioperative pain, cancer, chronic regional pain syndrome, abscess incision and drainage, limb fractures, closed reductions, and trauma.^19–32^ SDK at 0.3 mg/kg has been shown to provide effective pain control.^28,29,33^ To our knowledge, there have been no studies regarding the use of SDK (0.3 mg/kg) intravenous (IV) without the use of opiates for acute renal colic secondary to nephrolithiasis.

### Purpose of Study

The purpose of this clinical, prospective study was to determine the efficacy of utilizing SDK 0.3 mg/kg IV as an analgesic agent for renal colic in a convenience sample of ED patients. The authors’ hypothesis for this trial was that use of SDK would provide an effective supplemental non-opioid option for the treatment of acute renal colic in the ED with minimal adverse side effects.

## METHODS

### Study Design and Setting

This community-based study was a non-blinded prospective study testing the efficacy of an IV SDK dose of 0.3 mg/kg for the management of acute renal colic associated with nephrolithiasis in two EDs at a West-Michigan based health system. Before the study, the authors’ institutional review board approved the study protocol. Patients were enrolled upon written agreement using an approved informed consent according to institutional policy. This study was conducted in the Mercy Health ED campuses at the Hackley and Mercy facilities in Muskegon, Michigan. Combined, these campuses provide care to greater than 100,000 patients annually. The study population included a variety of ethnicities and ages. Enrollment period began in July 2016 and ended in February 2018.

### Selection of Participants

A convenience sample of eligible patients was obtained by board-certified emergency physicians, physician assistants, and emergency medicine residents using a standard renal colic protocol at the two ED study sites. Patients were offered participation based on clinical suspicion of nephrolithiasis after presenting with signs and symptoms of renal colic such as (but not limited to) flank, abdominal, groin or genital pain with nausea, vomiting, hematuria, and/or dysuria.

Inclusion criteria were: patients of all genders, race, and ethnicities between the ages 18-70. Exclusion criteria included: history of ketamine abuse, pregnancy, prior admission for kidney stones in the past 90 days, contraindications to study medications, severe respiratory disorders, schizophrenia, renal impairment, peptic ulcer disease, and recent gastrointestinal or intracranial hemorrhage. Patients who satisfied all study criteria were counseled utilizing an IRB approved informed consent document.

### Interventions

After consent was obtained, patients weighing greater than 50 kg received IV ketorolac 30 mg for analgesia (IV ketorolac 15 mg was administered if patients weighed less than 50 kg). At 30 minutes, pain was assessed using the 11-point verbal pain Numeric Rating Scale (NRS).^34^ If their pain was 5 or greater, IV ketamine 0.3 mg/kg was diluted in 50 mL of normal saline (NS) and infused over 10 minutes. This was defined as time zero. Study investigators then recorded pain scores using the 11-point verbal pain NRS as well as vital signs at 0, 30, 60, 90 and 120 minutes.

If patients were still experiencing pain after 30 minutes of the initial SDK dose, a second IV SDK dose was offered. After 90 minutes, if patients were still experiencing pain at an NRS score of 5 or greater, or requested additional medications, pain management was then implemented at the discretion of the ED provider. IV Midazolam 0.1 mg/kg was also available for patients who experienced anxiety or agitation. The data collection for each sample patient ended at time of their discharge from the ED. There were no follow up evaluations required. Discharge medications were left to the discretion of the treating provider.

### Measurements

Data were recorded using a paper data sheet with a patient identifying sticker. These data sheets were kept in the patients’ charts until all data collection was complete. Providers were asked to record vital signs, time, dosage, verbal pain NRS and any side effects experienced by patients. The data was de-identified by our lead investigator author (JG) when all data were collected and recorded. The de-identified data sheets were then placed in locked and collection boxes kept in the ED. These boxes were periodically emptied by study team members and manually uploaded into the Research Electronic Data Capture (REDCap) software for storage and future statistical analyses.^35^ Pursuant to our study design, patients were given the option to disenroll from the project at any time.

### Outcomes

The primary outcome measured was pain management after IV ketamine 0.3 mg/kg. Pain was assessed using an 11-point verbal pain NRS on a 0 to 10 scale. Pain scores and vital signs were measured at times 0, 30, 60, 90 and 120 minutes. Secondary outcomes measured included any adverse side effects.

### Analyses

Initial descriptive analyses were performed examining overall mean pain NRS scores for each intervention group, as well as among gender subgroups. Descriptive statistics were also generated for the number of recorded adverse events. Further analyses were carried out utilizing independent *t* tests and paired *t* tests to compare between intervention groups, and among initial relief of pain for ketamine with two-hour follow-up pain levels, respectively. All statistical analyses were carried out using the IBM Statistical Package for the Social Sciences (SPSS) version 25^36^ by the fifth author (SJW), with alpha cut-offs of 0.05 and a power of 0.80 specified.

## RESULTS

A total of n = 34 patients were enrolled in the study. Eight (23.5%) of these patients were given IV ketorolac with adequate pain relief and thus not requiring ketamine. A sample subgroup 26 (76.5%) patients were administered IV ketamine. There were n = 14 (41.2%) male subjects in the study, and n = 20 (58.8%) female subjects. Previous studies have established a 30% reduction in pain can be considered a clinically significant threshold for demarcating pain improvement.^37^ (Table 1)

Our primary endpoint was therefore to determine whether SDK met or exceeded this pain reduction goal. Overall, n = 23/34 (67.6%) of sample patients reported to have a clinically significant reduction in their pain score (> 30%). Of the patients who received SDK, n = 18/26 (69.2%) of study participants had a reduction in pain score > 30%. After receiving ketamine, pain was reduced from an initial mean pain score of 7.62/10 at time 0, to a pain score of 2.44 (SD = 0.78) at 30 minutes. (Table 2)

Patients who did not reach the authors’ observed 30% or higher pain reduction threshold had a mean pain score after 30 minutes of 6.88 (SD = 1.17). Independent *t* testing determined that these findings were significant, with a *t* = 3.16 and a *p* value = 0.004. In addition, the effect size for this difference was large, at 1.16 (95% CI 0.36 – 1.94). This suggests that not only did a large proportion of the patients receive benefits from IV ketamine in pain relief (n = 23/34 (69.2%), but also that the magnitude of pain relief conferred by receiving ketamine was substantial for this group.

We were unable to identify any statistically significant difference in reduction of pain NRS scores between genders. The average initial pain NRS score reported by patients who received IV SDK was 8.14 (SD = 0.63) for men and 7.50 (SD = 0.61) for women. The 30- minute pain scores in these groups were 2.63 (SD = 1.35) for men and 4.33 (SD = 0.91) for women. Independent *t* test analyses further revealed that this difference in 30-minute pain scores between the genders was not significant, with *t* = -1.047 and p = 0.306. This pain reduction was more pronounced two hours after receiving IV ketamine with mean pain for male participants of 2.38 (SD = 1.56) and a mean pain for female participants of 2.65 (SD = 0.65). Similarly, paired *t* test analyses revealed the difference in pain NRS scores two hours after the initial dose of IV ketamine was not significant across genders, *t* = -0.192, p = 0.850. (Table 3)

The most common adverse event experienced by sample patients was dizziness (17.5%), followed by feeling “high” (10.0%), and “drunk” (5.0%). Similar symptoms were found in previous studies examining IV SDK with reports of dizziness, nausea, and emesis.^19,20,33^ All reported symptoms resolved prior to discharge without use of midazolam. Several patients received anti-emetics for their nausea although data on the type and amount of medication were not collected. No patients experienced emergent reactions from SDK doses so there were no major adverse events in our study cohort. (Table 4)

Although our study was not adequately powered to examine the efficacy of IV ketorolac in pain reduction for renal colic, we did note a 30% or greater pain reduction with ketorolac alone in three of eight (38%) of patients. Initial average pain NRS scores in this group were 9.2 out of 10 which decreased to 6 out of 10 after receiving ketorolac. These findings were not, however, statistically significant (t = 1.78, p = 0.212). It is interesting to note that similar pain reduction has been documented in prior studies examining IV ketorolac for treatment of pain associated with renal colic.^9,38,39^

**Table:** Table 1 Sample Patient Demographics

	**Age (mean)**	**Gender**	**Weight (kg)**
**Total (n = 34)**	40.65 (SD = 14.87)	F 55.6% M 38.9%	93.0 (SD = 22.39)
**Ketamine (n = 26)**	41.69 (SD = 15.10)	F 69.2% M 30.8%	91.9 (SD = 21.69)
**Ketorolac (n = 8)**	37.25 (SD = 14.54)	F 25.0% M 75.0%	96.6 (SD = 25.79)

**Table:** Table 2 Mean Pain NRS Scores after Ketamine and Ketorolac

	**Original pain score**	**Pain score after ketamine**	**Pain score after ketorolac**	**Pain reduction 30%**	**Pain after 2 hrs**
**Total (n = 34)**	7.76 (SD = .437)	-	-	n = 23 (67.6%)	-
**Ketamine (n = 26)**	7.76 (SD = .437)	3.53 (SD = .661)	-	n =18 (69.2%)	2.56 (SD = 3.24)
**Ketorolac (n = 8)**	7.76 (SD = .437)	-	2.63 (SD = 3.99)	n = 5 (62.5%)	-

**Table:** Table 3 Differences in Mean Pain NRS Scores across Gender and Pain Reduction Threshold (30%)

	**Male (mean pain)**	**Female (mean pain)**	**p-value**
After Ketamine*	2.63 (SD = 1.35)	4.33 (SD = 0.91)	p = 0.31
2 Hours after Ketamine**	2.38 (SD = 1.56)	2.65 (SD = 0.65)	p = 0.85
After ketorolac*	3.33 (SD = 1.82)	0.50 (SD = 0.50)	p = 0.19
Pain Reduction 30%***	< 30%, 6.88 (SD = 1.17)	> 30%, 2.44 (SD = 0.78)	p = 0.004
**Effect size**	4.47 (95 % CI)		

*Independent T-test performed

**Paired T-test performed

***Pain reduction < of > 30% for the ketamine group (no gender differences examined)

**NRS** Numerical Rating Scale

**Table:** Table 4 Doses of Ketamine, Number of Adverse Events, and Additional Pain Medication, Relief with Ketorolac Alone

	**# of doses of ketamine**	**Adverse events**	**Additional medication**	**Relieved with ketorolac alone**
**Total (n = 34)**	1 dose (52.8%) 2 doses (19.4%)	n = 14 (38.9%)	n = 7 (19.4%)	-
**Ketamine (n = 26)**	-	n = 6 (23.1%)	-	-
**Ketorolac (n = 8)**	-	n = 8 (100%)	-	8/32 = 25%

## DISCUSSION

Our results demonstrate that SDK can be a viable therapeutic alternative to traditional treatment of renal colic in an ED setting. Several investigators have demonstrated the efficacy of low dose ketamine for a variety of patients presenting with pain complaints in the ED.^19,20,24,29,30,40^ During this study, we evaluated 34 patients who presented with signs and symptoms suggestive of renal colic and who received IV ketorolac followed by IV ketamine and found a statistically significant reduction in the verbal pain NRS scores that were independent of gender both initially and two hours after administration. Based on this finding, SDK can be considered an effective therapy option for the treatment of suspected renal colic in the ED.

Ketamine is a non-competitive antagonist at n-methyl-d-aspartic acid (NMDA) receptors with additional activity at mu opioid receptors. NMDA receptors are ligand-gated channels in the brain and spinal cord that bind the excitatory neurotransmitter glutamate. Current research suggests NMDA receptors are involved in pain transmission and modulation.^40^ The continuous binding of glutamate promotes the development of a hyperalgesia reflex arc, promoting pain via nociceptive neurons. Ketamine is thought to block this hyperalgesic pathway by antagonizing NMDA receptors, possibly explaining its unique analgesic properties.^28,29,41–43^ It can be administered IV, intramuscularly, intranasally, intraosseously, and by mouth. Ketamine has been shown to potentiate opioids when used simultaneously and decrease the amount required for analgesia.^42–45^ Ketamine-induced analgesia preserves respiratory reflexes, maintains cardiovascular stability and is not associated with hyperalgesia unlike increasing doses of opioids, making it a viable option for use in the ED for acute pain relief.^44,45^

Ketamine has fewer side effects (e.g., cardiopulmonary depression) than current analgesic medications used to treat renal colic. Most ED physicians avoid use of ketamine for fear of side effects, most notably emergence phenomenon featuring post-sedation hallucinations and agitation. Studies have found these events are dose-dependent and are unlikely to occur at sub-dissociative ketamine doses less than 1.0 mg/kg.^37^ The most common side effect of ketamine is dizziness.^27,29,32,44,46^ This was replicated in our study, with 17.5% of patients reporting this symptom.

The use of NSAIDs has been limited in patients with renal insufficiency, pregnancy, and history of gastrointestinal bleed, hence the reason alternatives are necessary for patients with renal colic. In contrast, with increasing doses, opioids can also cause life threatening respiratory depression and delirium.^28,43^ IV Ketamine has not been shown to display either of these adverse effects.

### Limitations

Our sample size, though adequately powered for statistical analysis, was small. This makes us wary of a Type II Error that might otherwise be disproven in a larger sample size. Our results also displayed large standard deviations for a portion of mean pain NRS scores, which might have been further narrowed (i.e., more accurate) in a larger sample. Likewise, our results were generated from a relatively small geographic area with a relatively homogenous patient population. Our enrollment criteria encompassed a wide range of ages (i.e., 18-70 years).

With a larger data set, further subgroup analyses stratified by age and gender might yield additional information to determine the ideal patient groups most appropriate for use of this medication. The prior administration of IV ketorolac may have contributed to decreases in pain after the patient had received ketamine as ketorolac has a duration of action of four to six hours. Future study groups may wish to investigate the utility of ketorolac and ketamine as monotherapy during inferiority / superiority studies as compared to opioid medications.

## CONCLUSIONS

In summary, our results indicate that IV SDK can be effective at treating pain associated with renal colic at 30 minutes and up to 120 minutes. Larger multicenter prospective studies are needed to confirm our results and ensure that they are generalizable to other ED settings and patient groups. We hope that these study results provide the basis for an expanded analgesic option in the acute management of renal colic. It behooves emergency physicians to consider non-opioid analgesia options in the midst of the current opioid epidemic and there is growing literature to support the use of SDK for acute pain management.

The review of this manuscript was coordinated by SMRJ Chief Editor William Corser.

### Conflict of Interest

The authors declare no conflict of interest.
